# Investigating potential disparities in clinical trial eligibility and enrollment at an NCI‐designated comprehensive cancer center

**DOI:** 10.1002/cam4.5933

**Published:** 2023-05-07

**Authors:** Monica A. Patel, Jennifer L. Shah, Floyd John Brinley, Paul H. Abrahamse, Christine M. Veenstra, Anne F. Schott

**Affiliations:** ^1^ Department of Medicine, Division of Hematology, Medical Oncology, and Palliative Care University of Wisconsin Madison Wisconsin USA; ^2^ Department of Radiation Oncology University of Michigan Ann Arbor Michigan USA; ^3^ Bio‐techne: ProteinSimple Inc San Jose California USA; ^4^ Department of Biostatistics University of Michigan Ann Arbor Michigan USA; ^5^ Department of Internal Medicine, Division of Hematology/Oncology University of Michigan Ann Arbor Michigan USA; ^6^ Institute for Healthcare Policy and Innovation University of Michigan Ann Arbor Michigan USA

**Keywords:** clinical trials, disparities, patient eligibility, trial enrollment

## Abstract

**Background:**

Although barriers to trial accrual are well‐reported, few studies have explored trial eligibility and trial offers as potential drivers of disparities in cancer clinical trial enrollment.

**Methods:**

We identified patients with gastrointestinal (GI) or head/neck (HN) malignancies who were seen as new patients at the University of Michigan Health Rogel Cancer Center in 2016. By exhaustive review of the electronic medical record, we assessed the primary outcomes: (1) eligibility for, (2) documented offer of, and (3) enrollment in a clinical trial. All 41 of the clinical trials available to these patients were considered. Independent variables included clinical and non‐clinical patient‐related factors. We assessed associations between these variables and the primary outcomes using multivariable regression.

**Results:**

Of 1446 patients, 43% were female, 15% were over age 75, 6% were Black. 305 (21%) patients were eligible for a clinical trial. Among eligible patients, 154 (50%) had documentation of a trial offer and 90 (30%) enrolled. Among the GI cohort, bivariate analyses demonstrated that older age was associated with decreased trial eligibility. Bivariate analyses also demonstrated that Black race was associated with increased trial offer. After adjustment, patients 75 or older were less likely to be eligible for a clinical trial in the GI cohort; however, we found no significant associations between race and any of the outcomes after adjustment. Among eligible GI patients, we found no significant associations between non‐clinical factors and enrollment. Among the HN cohort, bivariate analyses demonstrated that female sex, older age, Black race, and unpartnered marital status were associated with decreased likelihood of trial offer; however, we found no significant associations between race, age, and marital status and any of the outcomes after adjustment. We found no significant associations between non‐clinical factors and eligibility after adjustment; however, women were less likely to be offered and to enroll in a clinical trial in the HN cohort.

**Conclusion:**

Factors associated with eligibility, documented offer, and enrollment differed between disease site cohorts at our institution. Future work is needed to ensure the equitable inclusion of women and elderly patients in clinical trials.

## Abstract 2

A thorough review assessing the clinical trial eligibility of individual cancer patients seen at an academic center demonstrates that only 21% of patients were eligible for any trials, and only half of these eligible patients had documented evidence of clinical trial offer. Specific demographic groups were less likely to receive a trial offer. Interventions in the clinical encounter are needed to improve recognition of trial eligibility and facilitate trial offer if we hope to improve accrual to cancer clinical trials.

## 1 INTRODUCTION

Although clinical trials are a key component of high‐quality cancer care, patient enrollment in clinical trials is low: less than 10% of adults with cancer participate in clinical trials.^1^, ^2^ Furthermore, the results of clinical trials may not be representative of the general population because, among the small percentage of patients who enroll in clinical trials, there are few women and minority participants.^3^, ^4^, ^5^, ^6^ In 1993, the National Institutes of Health passed the Revitalization Act to address disparities in clinical trial enrollment, specifically focusing on the inclusion of women and minorities.^7^ Since that time, however, multiple studies have shown that disparities continue to persist with women, minorities, and elderly patients being underrepresented in clinical trials relative to the general population.^3^, ^4^, ^5^, ^6^, ^8^, ^9^ The disproportionate enrollment of these groups limits the generalizability of trial results to the entire population affected by the disease.

The process of recruiting and enrolling subjects into clinical trials is complex, and many barriers to accrual have been identified. A systematic review from 2008 reported that older age, lower socioeconomic status, being a racial or ethnic minority, and increased comorbidities were barriers to clinical trial participation.^4^ However, patients' eligibility for clinical trials and eligible patients receiving trial offers in a clinical encounter have not been closely examined. Even when patients are eligible for clinical trials, they may not be offered trials by their physicians or choose to enroll. In a survey study of 5499 patients with cancer, 40% of patients reported a clinical trial discussion with their physician, but less than half (45%) of these discussions led to a physician offering a trial to a patient. Moreover, only half (51%) of patients offered a trial actually enrolled for an overall 9% clinical trial participation rate.^10^

Given low clinical trial accrual and disparities in clinical trial enrollment, it is critical to understand the potential limitations related to clinical trial eligibility criteria and making trial offers as well as barriers to enrollment in order to improve equity in clinical trial participation. At the University of Michigan Health Rogel Cancer Center, we recognized what seemed to be a disparity in enrollment in cancer clinical trials by sex, with a smaller proportion of women enrolling in clinical trials compared to the proportion of women among all new patients seen in our cancer center. This disparity seemed most apparent in gastrointestinal (GI) and head/neck (HN) cancers. Since trial participation is a multifactorial problem differing by practice environment, we sought to understand this and other potential disparities at our institution by systematically reviewing all new patients with GI and HN malignancies seen in 2016 to investigate factors related to (1) eligibility in a clinical trial, (2) documented offer of a clinical trial, and (3) enrollment in a clinical trial.

## 2 METHODS

### 2.1 Study population

The study population consisted of all analytic (new presentation) patient cases with GI and HN malignancies in the year 2016 based on institutional tumor registry data. We chose 2016 as this was the most recent year that the tumor registry was complete at the time of study initiation. GI patients had cancers of the anus, colon, liver, pancreas, rectum, small intestine, stomach, or other digestive organ. HN patients had cancers of the mucosal tract (oral cavity, oropharynx, larynx, hypopharynx, nasopharynx), salivary glands, or thyroid. Adult patients age 18 or older were included. All stages from 0 to IV were included. The study was approved by the University of Michigan Institutional Review Board.

### 2.2 Outcomes

We assessed three primary outcomes in the study population: (1) eligibility for a clinical trial, (2) documented offer of a clinical trial, and (3) enrollment in a clinical trial. We used the University of Michigan Health Rogel Cancer Center clinical trials database to determine all clinical trials open to enrollment in 2016 for patients with GI and HN malignancies. We then did a thorough and meticulous review of the electronic medical record (EMR) to assess the primary outcomes. Screening logs were not available. We obtained the clinical trial protocol for each open trial, and we assessed eligibility of each patient for each trial based on the inclusion and exclusion criteria listed in the protocol for each trial. We ensured that the trials being considered were open to accrual on the dates of patient evaluation in clinic. We specifically abstracted data from the problem list, provider notes, labs results, and imaging results to assess eligibility. Typical eligibility criteria in the trial protocols included age, stage, histologic confirmation, ECOG PS, adequate hematologic function, adequate hepatorenal function, absence of prior therapy that would limit current therapy on trial, absence of prior cancer within 3 years, current participation in another trial, and pregnancy/breastfeeding. Other eligibility criteria were variable based on the type and stage of cancer being investigated. We determined clinical trial offer and enrollment by EMR review, specifically including notes of providers in oncologic specialties and research coordinators. Trial enrollment was confirmed by cross referencing the EMR with documentation in the cancer center clinical trials database, in which patients are required to be registered in real‐time in order to be enrolled to a study. Trial enrollment necessarily implied trial eligibility and trial offer. Given concerns about the potential for trial offers that were made but not documented if a patient chose not to enroll, the subgroup of patients who were eligible for a trial was used as the denominator for analyses of trial offer and trial enrollment.

### 2.3 Independent variables

We considered both clinical and non‐clinical patient‐level variables that may be associated with trial eligibility, offer, and enrollment. Clinical patient‐level variables included age, Charlson Comorbidity Index (CCI), second primary cancer, primary site, and American Joint Committee on Cancer (AJCC) Stage. Non‐clinical patient‐level variables included sex, race, marital status, having children, employment status at diagnosis, and primary payer at diagnosis. Given that marital status, number of children, employment status, and primary payer should not impact eligibility, these were not assessed for clinical trial eligibility.

### 2.4 Statistical analysis

Using chi‐square tests, we evaluated bivariate associations between each primary outcome and the independent variables. We used multivariable logistic regression models to evaluate our outcomes. All statistical tests were two‐sided. *p* values ≤0.05 were considered significant. For the multivariable models, we also ran reduced models with fewer covariates to examine if overfitting would be a factor and found similar results. Analyses were conducted with SAS 9.4 (Carey).

## 3 RESULTS

### 3.1 Gastrointestinal cohort

We identified 821 patients with GI malignancies and 625 patients with HN malignancies who were included as analytic cases in the tumor registry in 2016. Characteristics of the GI cohort by independent variables assessed are described in Table 1. Of 821 patients, 40% were female, 48% were age 65 or older, and 87% were White. 45% had Stage III/IV disease, and 29% had a CCI of <5.

**TABLE 1:** Characteristics of the entire patient sample (*N* = 1446).

Characteristic	GI (*n* = 821), *n* (%)	HN (*n* = 625), *n* (%)
Gender		
Female	330 (40)	290 (46)
Male	491 (60)	335 (54)
Age		
<65	424 (52)	420 (69)
65–74	250 (30)	133 (19)
≥75	147 (18)	72 (12)
Race		
White	715 (87)	574 (92)
Black	62 (7)	18 (3)
Asian	19 (2)	3 (3)
Other	18 (2)	10 (2)
Missing/unknown	7 (1)	20 (1)
Marital status		
Not married	291 (35)	224 (36)
Married/partnered	519 (63)	390 (62)
Missing/unknown	17 (2)	11 (2)
Children		
0	133 (16)	90 (14)
1 or more	673 (81)	444 (71)
Missing/unknown	21 (3)	91 (15)
Employment status		
Full‐time	248 (30)	251 (49)
Part‐time	39 (5)	23 (5)
Retired	366 (44)	163 (32)
Not working	154 (19)	69 (14)
Missing/unknown	20 (2)	3 (1)
Primary payer		
Private	290 (35)	263 (42)
Medicare	383 (46)	249 (40)
Medicaid/state	88 (11)	99 (16)
Other	56 (7)	5 (1)
Missing/unknown	10 (1)	9 (1)
CCI		
<5	242 (29)	394 (63)
5–7	343 (41)	189 (30)
≥8	235 (29)	42 (7)
Missing/unknown	1 (1)	2 (<1)
Second primary cancer		
Yes	67 (8)	135 (22)
No	753 (91)	490 (78)
Primary site: GI		
Colorectal	289 (35)	N/A
Hepatobiliary	389 (47)	
Other	143 (17)	
Missing/unknown	6 (1)	
Primary site: HN		
Mucosal	N/A	370 (59)
Salivary		49 (8)
Thyroid		206 (33)
AJCC stage		
0/I	175 (21)	227 (36)
II	180 (22)	49 (8)
III	151 (18)	82 (13)
IV	221 (27)	251 (40)
Missing/unknown	94 (12)	16 (3)

Abbreviation: CCI, Charlson Comorbidity Index.

**FIGURE 1:**
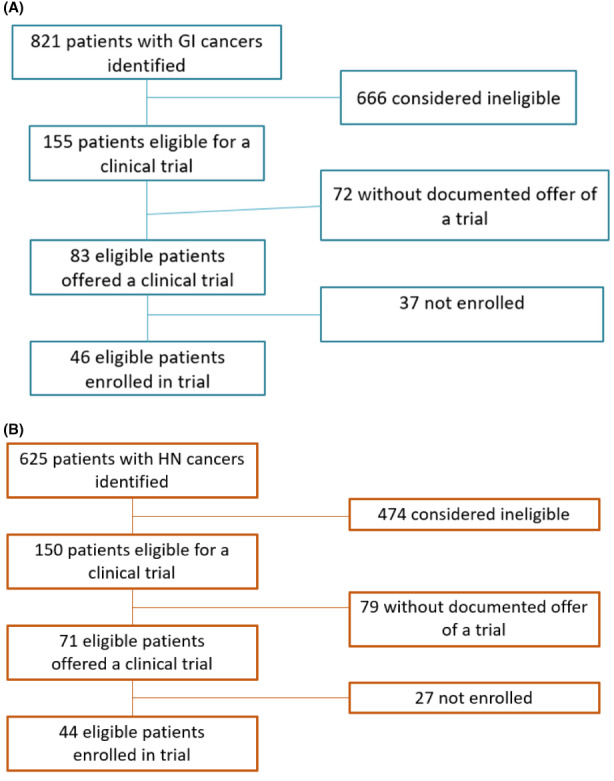
(A) Patients who were eligible, offered, and enrolled in a clinical trial from GI cohort. (B) Patients who were eligible, offered, and enrolled in a clinical trial from the HN cohort.

A total of 23 clinical trials were available to GI patients; 8 for colorectal primary, 12 for hepatobiliary/pancreatic primary, 3 for other GI cancer types. Based on detailed chart abstraction to check inclusion and exclusion criteria, 155 (19%) patients were eligible for a clinical trial. Of those, 83 (54%) had documentation of a trial being offered by the provider, and 46 (30%) enrolled in a clinical trial (Figure 1A). Overall, 6% of the entire GI cohort were trial enrollees.

Bivariate analyses of eligibility for a clinical trial, offer of a clinical trial, and enrollment in a clinical trial were conducted for patients with GI malignancies and are demonstrated in Table S1. Older age (*p* < 0.01), second primary cancer (*p* < 0.01), and early‐stage disease (*p* < 0.01) were associated with decreased clinical trial eligibility. Black race (*p* = 0.05), having 1 or more children (*p* = 0.01), and medicare insurance (*p* = 0.01) were significantly associated with increased documentation of trial offer. Full‐time employment was associated with decreased trial enrollment (*p* = 0.04). The primary site of disease (hepatobiliary/pancreatic) was significantly associated with all primary outcomes (*p* < 0.01 for all outcomes).

For the GI cohort, multivariable analyses are demonstrated in Table 2. Older age (OR 0.32, 95% CI 0.14–0.78 for age ≥75 vs. age <65), increased CCI (OR 0.59, 95% CI 0.35–0.99 for CCI 5+ vs. <5), second primary cancer (OR 0.19, 95% CI 0.04–0.8 for yes vs. no), and early‐stage disease (OR 0.19, 95% CI 0.09–0.42 for Stage 0/1 vs. IV; OR 0.73, 95% CI 0.38–1.4 for Stage II vs. IV) were associated with a decreased odds of clinical trial eligibility. Having no children (OR 4.99, 95% CI 1.29–19.35 for 1 or more vs. 0) and an increased CCI (OR 0.19, 95% CI 0.06–0.66 for CCI 5+ vs. <5) were associated with a decreased odds of clinical trial offer. Early‐stage disease (OR 0.73, 95% CI 0.12–4.53 for Stage II vs. IV) was associated with a decreased odds of clinical trial enrollment. Primary site of hepatobiliary/pancreatic primary was associated with increased odds of all outcomes, including eligibility (OR 2.53, 95% CI 1.58–4.06 for hepatobiliary/pancreatic vs. colorectal), offers (OR 6.59, 95% CI 2.04–21.28 for hepatobiliary/pancreatic vs. colorectal), and enrollment (OR 6.09, 95% CI 1.58–23.44 for hepatobiliary/pancreatic vs. colorectal) in clinical trials.

**TABLE 2:** Multivariable model of eligibility, documented offer, and enrollment, among patients with GI malignancy.

	Eligibility (*n* = 821)		Documented offer, among patients eligible for trial (*n* = 155)		Enrollment, among patients eligible for trial (*n* = 155)	
Characteristic	Odds ratio (95% CI)	*p*	Odds ratio (95% CI)	*p*	Odds ratio (95% CI)	*p*
Gender						0.26
Male	Ref	0.50	Ref	0.93	Ref	
Female	0.87 (0.58–1.31)		1.05 (0.40–2.74)		0.56 (0.21–1.53)	
Age		<0.01		0.50		0.94
<65	Ref		Ref		Ref	
65–74	1.36 (0.85–2.17)		2.10 (0.54–8.15)		0.80 (0.22–2.94)	
≥75	0.32 (0.14–0.78)		0.85 (0.11–6.50)		0.79 (0.09–6.79)	
Race		0.44		0.17		0.78
White	Ref		Ref			
Asian	0.51 (0.11–2.46)		‐		Ref	
Black	0.54 (0.23–1.28)		26.27 (1.48–466.59)		‐	
Other	0.8 (0.21–3.09)				2.21 (0.30–16.17)	
Marital status	N/A			0.08		0.84
Not married			Ref		Ref	
Married/partnered			2.46 (0.90–6.73)		1.11 (0.41–2.96)	
Children	N/A			0.02		0.64
0			Ref		Ref	
1 or more			4.99 (1.29–19.35)		1.37 (0.37–5.14)	
Employment status	N/A			0.81		0.08
Full‐time			Ref		Ref	
Part‐time			1.30 (0.19–8.96)		2.76 (0.40–19.05)	
Retired			0.63 (0.20–1.99)		1.47 (0.49–4.4)	
Not working			0.82 (0.21–3.25)		0.19 (0.04–1.02)	
Primary payer	N/A			0.40		0.82
Private			Ref		Ref	
Medicaid			1.60 (0.35–7.41)		1.12 (0.22–5.64)	
Medicare			3.21 (0.81–12.7)		1.43 (0.35–5.95)	
Other			1.98 (0.27–14.63)		2.29 (0.37–14.12)	
Charlson Comorbidity Index		0.03		0.03		0.85
<5	Ref		Ref		Ref	
6–7	0.6 (0.35–1.01)		0.18 (0.05–0.63)		1.37 (0.41–4.59)	
8+	0.35 (0.16–0.77)		0.18 (0.02–1.60)		1.70 (0.21–13.62)	
Second primary cancer		0.02		0.97		0.73
No	Ref		Ref		Ref	
Yes	0.19 (0.04–0.8)		0.05 (0.01–99.0)		0.54 (0.02–17.85)	
Primary site		<0.01		<0.01		0.01
Colorectal	Ref		Ref		Ref	
Hepatobiliary/pancreatic	2.53 (1.58–4.06)		6.59 (2.04–21.28)		6.09 (1.58–23.44)	
Other	0.41 (0.18–0.92)		0.10 (0.01–1.64)		0.49 (0.04–6.24)	
AJCC stage		<0.01		0.07		0.05
0/I	0.19 (0.09–0.42)		1.05 (0.14–8.08)		1.52 (0.22–10.66)	
II	0.73 (0.38–1.4)		0.37 (0.06–2.37)		0.73 (0.12–4.53)	
III	1.09 (0.57–2.09)		2.18 (0.33–14.57)		4.74 (0.68–33.04)	
IV	Ref		Ref		Ref	

### 3.2 Head/neck cohort

Characteristics of the HN cohort by independent variables assessed are described in Table 1. Of 625 patients, 46% were female, 31% were age 65 or older, and 92% were White. Over half of patients (53%) had Stage III/IV disease and 63% had a CCI of <5.

A total of 18 clinical trials were available to HN patients: 10 for newly diagnosed mucosal primary, 5 for recurrent/metastatic mucosal primary, 1 for nasopharynx primary, 1 for salivary primary, and 1 for thyroid primary. Based on detailed chart abstraction to check inclusion and exclusion criteria, 150 (24%) patients were eligible for a clinical trial, of which 143 had a mucosal primary and 7 had a salivary primary. Of eligible patients, 71 (47%) had documentation of trial offer, and 44 (29%) enrolled in a clinical trial (Figure 1B). No patients with a thyroid primary were eligible for the one clinical trial open for thyroid malignancies. There were no trial offers or enrollments among the eligible patients with salivary cancer. Overall, 31% of eligible patients with a mucosal primary enrolled into a trial. This represents 29% of all eligible HN patients and 7% of the entire HN cohort.

Bivariate analyses for the independent variables associated with the outcomes were conducted for the HN cohort and are demonstrated in Table S2. Female sex (*p* < 0.01), older age (*p* < 0.01), second primary cancer (*p* < 0.01), non‐mucosal primary (*p* < 0.01), and early‐stage disease (*p* < 0.01) were associated with decreased trial eligibility. Female sex (*p* < 0.01), older age (*p* = 0.03), Black race (*p* = 0.03), not being married (*p* = 0.03), being retired (*p* < 0.01), Medicare insurance (*p* = 0.01), and salivary primary (*p* = 0.01) were significantly associated with decreased likelihood of trial offer. Female sex (*p* < 0.01) and being retired (*p* = 0.03) were associated with decreased likelihood of trial enrollment.

Multivariable analyses for the HN cohort are shown in Table 3. Second primary cancer (OR 0.31, 95% CI 0.15–0.61 for yes vs. no), non‐mucosal primary (OR 0.3, 95% CI 0.11–0.81 for salivary vs. mucosal), and lower stage disease (OR 0.21, 95% CI 0.09–0.51 for Stage II vs. IV) were associated with decreased odds of trial eligibility. Being retired or not working was associated with decreased odds of trial offer (OR 0.15, 95% CI 0.03–0.71 for retired vs. full‐time; OR 0.39, 95% CI 0.07–2.07 for not working vs. full‐time). Female sex was associated with decreased odds of trial offer (OR 0.2, 95% CI 0.05–0.73 for female vs. male) and trial enrollment (OR 0.1, 95% CI 0.02–0.51 for female vs. male).

**TABLE 3:** Multivariable model of eligibility, documented offer, and enrollment, among patients with HN malignancy.

	Eligibility (*n* = 625)		Documented offer, among patients eligible for trial (*n* = 150)		Enrollment, among patients eligible for trial (*n* = 150)	
Characteristic	Odds ratio (95% CI)	*p*	Odds ratio (95% CI)	*p*	Odds ratio (95% CI)	*p*
Gender						
Male	Ref	0.80	Ref	0.02	Ref	0.01
Female	0.93 (0.53–1.63)		0.20 (0.05–0.73)		0.10 (0.02–0.51)	
Age						
<65	Ref	0.33	Ref	0.68	Ref	0.32
65–74	1.42 (0.7–2.86)		2.11 (0.40–11.12)		3.58 (0.62–20.88)	
≥75	0.74 (0.29–1.89)		1.77 (0.13–24.19)		4.64 (0.34–63.92)	
Race						
White	Ref	0.83	Ref	0.99	Ref	0.99
Asian	3.34 (0.19–59.71)		0.90 (0.04–21.77)		0.84 (0.31–22.90)	
Black	1.2 (0.27–5.36)		‐		‐	
Other	1.36 (0.35–5.33)		‐		1.20 (0.18–7.89)	
Marital status	N/A					
Not married			Ref	0.16	Ref	0.74
Married/partnered			2.51 (0.70–8.95)		0.81 (0.23–2.85)	
Children	N/A					
0			Ref	0.94	Ref	0.27
1 or more			1.05 (0.21–5.45)		0.42 (0.09–1.97)	
Employment status	N/A					
Full‐time			Ref	0.03	Ref	0.46
Part‐time			‐		‐	
Retired			0.15(0.03–0.71)		0.31 (0.06–1.58)	
Not working			0.39 (0.07–2.07)		1.17 (0.21–6.40)	
Primary payer	N/A					
Private			Ref	0.32	Ref	0.39
Medicaid			1.58 (0.33–7.65)		0.53 (0.12–2.23)	
Medicare			0.31 (0.07–9.75)		0.25 (0.05–1.23)	
Other			‐		‐	
Charlson Comorbidity Index						
<5	Ref	0.13	Ref	0.40	Ref	0.98
6–7	0.54 (0.28–1.05)		2.54 (0.66–9.75)		1.12 (0.30–4.15)	
8+	0.43 (0.14–1.29)		1.66 (0.07–1.48)		1.16 (0.07–18.61)	
Second primary cancer						
No	Ref	<0.01	Ref	0.69	Ref	0.56
Yes	0.31 (0.15–0.61)		1.40 (0.27–7.37)		1.71 (0.29–10.21)	
Primary site						
Mucosal	Ref	0.05	Ref	0.95	Ref	0.94
Salivary	0.3 (0.11–0.81)		0.10 (0.01–99.0)		0.01 (0.01–99.0)	
Thyroid	999 (0–999)		‐		‐	
AJCC stage						
0/I	‐	<0.01	‐	0.36	‐	0.62
II	0.21 (0.09–0.51)		0.17 (0.02–1.98)		‐	
III	0.76 (0.38–1.51)		0.99 (0.28–3.59)		0.52 (0.14–1.95)	
IV	Ref		Ref		Ref	

*Note*: Because they do not impact clinical trial eligibility, marital status, children, employment status, and primary payer were not included as covariates in the multivariable model of clinical trial eligibility.

## 4 DISCUSSION

We observed a potential sex disparity in the trial enrollment data at our institution and sought to identify factors associated with eligibility, offers, and enrollment with the goal of defining targetable barriers to inform development of interventions to improve trial accrual overall. In our study of patients with newly diagnosed GI and HN malignancies seen at the University of Michigan Health Rogel Cancer Center in 2016, we found among the entire cohort that 21% were eligible for a clinical trial, 11% had documentation of trial offer, and 6% enrolled in a clinical trial. This finding is consistent with prior data demonstrating that less than 10% of adult patients with cancer participate in clinical trials.^1^, ^2^

Our study identifies eligibility based on inclusion and exclusion criteria as a major limitation to trial accrual. Previous studies have demonstrated that approximately half of patients do not have a clinical trial available to them.^1^, ^2^ At our institution, we found that there was a lack of trials for rare cancers, including salivary, and anaplastic thyroid cancers. Reasons for ineligibility in both the GI and HN cohorts included lab abnormalities, ECOG performance status, disease stage, prior history of malignancy, and other criteria that were protocol‐specific. A goal of increasing trial participation at large implicates prioritizing the development and opening of trials to a wider range of patients, diagnoses, and disease stages. Continual use of traditional inclusion and exclusion criteria can systemically and perpetually exclude underrepresented patient populations. For example, recent data from Virginia Commonwealth University Health demonstrated that fewer Black patients were eligible for pancreas cancer clinical trials when compared to White patients, largely due to low albumin levels and co‐existing infectious disease.^11^ Importantly, we did not find significant disparities in eligibility in either the HN or GI cohorts by sex or race in multivariable analysis.

However, in the GI cohort, bivariate and multivariable analyses demonstrated that patients aged 75 or over were less likely to be eligible for a clinical trial. Multiple studies have demonstrated that elderly patients are not well represented in cancer clinical trials, and this has been identified as a health disparity.^3^, ^8^ Given that almost half of the GI cohort was aged 65 or older, this finding in our study emphasizes that strict eligibility criteria surrounding age and co‐existing age‐related medical conditions should be limited to ensure equitable inclusion of elderly patients in clinical trials. In addition, elderly patients should be represented in clinical trials in order to safely assess the efficacy and toxicities of treatment among all age groups. This is consistent with recent Food and Drug Administration (FDA) guidance on the inclusion of older patients in cancer clinical trials, especially those above the age of 75.^12^

Other clinical factors associated with decreased clinical trial eligibility, both in bivariate and multivariable analyses, among the GI and HN cohorts were having a second primary cancer and early‐stage disease. These findings are not surprising given generalized lack of clinical trial availability for early‐stage patients and second primary cancer being a common exclusion criterion.

Documented trial offer to eligible patients was also a major limitation to enrollment in our cohort with 54% of eligible GI patients and 47% of eligible HN patient having a documented trial offer. We recognize that we were only able to assess trial offers that were documented in the patient's chart, and we therefore likely missed some trial offers that occurred in the setting of a clinical encounter but were not recorded in the chart. We did not specifically collect data on the number of patients who enrolled in a trial who did not have documentation of trial offer in their charts, as enrollment implies offer of a trial. Additionally, some factors that impact eligibility, such as ECOG performance status, were not routinely documented and could limit trial offer. Other aspects of a patient's profile, such as logistical and financial circumstances, are also not well captured in the EMR but can impact trial offer. In the GI cohort, having one or more children was associated with increased trial offer, perhaps reflecting children implicating more social support and positively contributing to provider‐assessed trial suitability and, therefore, trial offer. In the HN cohort, those who were retired or not working were less likely to have documentation of trial offer, potentially indicating non‐working status reflecting poor performance status and negatively affecting trial offer. These non‐clinical factors are also confounded by logistical and financial considerations in ways that even screening logs would be unlikely to clarify.

Among the GI cohort, bivariate analyses demonstrated that Black race was associated with increased trial offer. Among the HN cohort, bivariate analyses demonstrated that Black race was associated with decreased trial offer. Importantly, after adjustment we did not find significant disparities by race in trial offer in either the HN or GI cohorts. Regardless, these findings highlight the need to recognize implicit bias and to develop interventions, such as online training programs, to address potential biases that can influence clinical trial offer.^13^ We do note the low percentage of racial minorities in our study reflective of the patient population at our institution. Given limitations of trial eligibility criteria, this meant an even smaller number of minority patients who were eligible for a trial. With the limited representation of minority patients in our study, these results should be interpreted with caution. However, this highlights the systemic bias of cancer clinical trials largely being conducted at tertiary academic centers and designed by investigators at these institutions which often serve a less diverse patient population.

Notably, however, the majority (58%) of patients with documented trial offer did enroll, which is also consistent with published data.^14^ Physician surveys and data from the National Cancer Institute's Community Cancer Centers Program Clinical Trial Screening and Accrual Log indicate that provider‐based factors, such as time constraints, lack of comfort in discussing trials with patients, and physician bias, impede clinical trial offers.^15^, ^16^, ^17^, ^18^ Improving trial accrual necessitates interventions to facilitate trial offers in the clinical setting. Further studies are needed to understand this issue in greater detail; this could include qualitative work to consider which patients are offered a clinical trial and how physicians decide to make a trial offer.

We intentionally assessed enrollment among eligible patients due to the limitations of documented trial offer and inherent assumptions in using this as a denominator. In the GI cohort, we found no significant associations between non‐clinical factors and clinical trial enrollment. In the HN cohort, women were less likely to enroll on a trial. Though we attempted to control for factors that may align with sex, it is possible that uncaptured social determinants are contributing to these results. Factors pertaining to transportation and income, for example, may affect trial enrollment and may vary by sex, but were not included in our study. Communication in the doctor‐patient relationship has also been shown to vary by sex,^19^, ^20^ and this may also affect clinical trial recruitment and enrollment. It is possible that the subconscious impact of non‐clinical factors and implicit bias among physicians may also contribute to these findings. This issue has been most aptly demonstrated by a study of physicians evaluating standardized recorded interviews of patients with chest pain; female and Black patients were less likely to be referred for cardiac catheterization than male and white patients.^21^ The cancer community must develop interventions and approaches to reduce bias in evaluating patients for clinical trials.

There are several limitations to our study that warrant mention. We recognize that the data derived in our study are from relatively small numbers at a single tertiary cancer center and may not apply to the overall population's eligibility or candidacy for clinical trials. However, by focusing on our institution alone, we were able to perform an exhaustive chart review and provide a granular level of detail that may not have been possible on a larger scale. In addition, at our institution, the process for screening for trial eligibility is variable among multidisciplinary disease‐site‐focused groups and may include physician screening as well as screening in tumor board discussions. HN patients at our institution are reviewed at tumor board and screened for clinical trial eligibility. For GI patients, there is no standardized process for GI patients, and screening is done by individual providers at the time of the clinical encounter. Furthermore, there are some factors that providers take into consideration when assessing a patient for trial eligibility, such as provider‐perceived compliance, reliability, and health literacy of a patient, that are not routinely documented and are not part of the formal eligibility criteria but may contribute to provider bias and influence providers' decision to offer clinical trials. One possible solution is development of a standardized assessment of non‐clinical factors or a standardized institutional process to help screen patients for clinical trials and facilitate enrollment. Further study is needed to better elucidate these factors and inform potential interventions.

## 5 CONCLUSION

Very few patients participate in cancer clinicals trials, and there are significant disparities among trial participants.^3^, ^8^ Our study uniquely evaluates trial eligibility, documented offers, and enrollment at the individual patient level and demonstrates that non‐clinical factors, including age and sex, continue to be barriers to clinical trial participation. Future work is needed to broaden eligibility, facilitate trial offers, and ensure equitable participation in cancer clinical trials.

## AUTHOR CONTRIBUTIONS

**Monica A. Patel:** Conceptualization (equal); data curation (lead); funding acquisition (lead); investigation (lead); methodology (lead); project administration (lead); writing – original draft (lead); writing – review and editing (lead). **Jennifer Shah:** Conceptualization (equal); data curation (lead); investigation (lead); methodology (lead); project administration (lead); writing – original draft (lead); writing – review and editing (lead). **Floyd John Brinley:** Data curation (equal); writing – review and editing (equal). **Paul Abrahamse:** Formal analysis (lead); writing – review and editing (equal). **Christine M Veenstra:** Conceptualization (lead); methodology (lead); supervision (lead); writing – review and editing (equal). **Anne Francis Schott:** Conceptualization (lead); funding acquisition (lead); methodology (lead); supervision (lead); writing – review and editing (equal).

## CONFLICT OF INTEREST STATEMENT

The authors have no conflict of interest to declare.

## FUNDING STATEMENT

Dr. Patel and this work were funded by the Ruth L. Kirschstein Institutional Research Training Grant under Award Number T32CA9357‐37. Any opinions, findings, and conclusions expressed in this material are those of the author(s). Research reported in this publication was also supported by the National Cancer Institutes of Health under Award Number P30CA046592. The content is solely the responsibility of the authors and does not necessarily represent the official views of the National Institutes of Health.

## Supporting information


**Supplemental Table 1:** Bivariate Analyses of Trial Eligibility, Documented Offer, and Enrollment, Among Patients with GI Malignancy
Supplemental Table 2: Bivariate Analyses of Trial Eligibility, Documented Offer, and Enrollment, Among Patients with HN Malignancy

## Data Availability

The data that support the findings of this study are available on request from the corresponding author. The data are not publicly available due to privacy or ethical restrictions.
