# Supplementary material for: Investigating potential disparities in clinical trial eligibility and enrollment at an NCI‐designated comprehensive cancer center
Source: Cancer Med. 2023 May 7;12(11):12802–12. doi: 10.1002/cam4.5933 (PMC10278521; doi:10.1002/cam4.5933)
Supplement: Supplementary file 1 — Supplemental Table 1: Bivariate Analyses of Trial Eligibility, Documented Offer, and Enrollment, Among Patients with GI Malignancy Supplemental Table 2: Bivariate Analyses of Trial Eligibility, Documented Offer, and Enrollment, Among Patients with HN Malignancy [file CAM4-12-12802-s001.docx]

| Supplemental Table 1: Bivariate Analyses of Trial Eligibility, Documented Offer, and Enrollment, Among Patients with GI Malignancy | | | | | | | | | |
| --- | --- | --- | --- | --- | --- | --- | --- | --- | --- |
|  | Eligibility (N=821) | | | Documented Offer, Among Patients Eligible for Trial (N=155) | | | Enrollment, Among Patients Eligible for Trial (N=155) | | |
| **Characteristic** | **Eligible**  **N(%)** | **Not eligible**  **N(%)** | **P** | **Documented**  **N(%)** | **Not Documented**  **N(%)** | **P** | **Enrolled** | **Not Enrolled** | **P** |
| Gender  Female  Male | 55 (17)  100 (20) | 275 (83)  391 (80) | 0.18 | 29 (53)  54 (54) | 26 (47)  46 (46) | 0.88 | 13 (24)  33 (33) | 42 (76)  67 (67) | 0.22 |
| Age  <65  65-74  ≥75 | 87 (21)  61 (24)  7 (5) | 337 (79)  189 (76)  140 (21) | <0.01 | 41 (47)  39 (64)  3 (43) | 46 (53)  22 (36)  4 (57) | 0.11 | 22 (48)  22 (48)  2 (4) | 65 (60)  39 (36)  5 (4) | 0.37 |
| Race  White  Black  Asian  Other | 139 (20)  7 (11)  2 (11)  3 (17) | 576 (80)  55 (89)  17 (89)  15 (83) | 0.33 | 74 (52)  6 (86)  0 (0)  3 (100) | 67 (48)  1 (14)  2 (100)  0 (0) | 0.05 | 42 (30)  3 (43)  0 (0)  1 (33) | 97 (70)  4 (57)  2 (100)  2 (67) | 0.71 |
| Marital status  Not Married  Married/Partnered | N/A | N/A | - | 20 (44)  63 (57) | 25 (56)  47(43) | 0.15 | 12 (27)  34 (31) | 33 (73)  76 (69) | 0.60 |
| Children  0  1 or more | N/A | N/A | - | 6 (27)  76 (58) | 16 (73)  56 (42) | 0.01 | 5 (23)  41 (31) | 17 (77)  91 (69) | 0.43 |
| Employment status  Full-time  Part-time  Retired  Not working | N/A | N/A | - | 27 (48)  4 (44)  37 (58)  14 (54) | 29 (52)  5 (56)  27 (42)  12 (46) | 0.72 | 14 (25)  3 (33)  26 (41)  3 (12) | 41 (75)  6 (67)  38 (59)  23 (88) | 0.04 |
| Primary payer  Private  Medicare  Medicaid  Other | N/A | N/A | - | 27 (44)  43 (68)  7 (33)  6 (67) | 35 (56)  20 (32)  14 (67)  3 (33) | 0.01 | 13 (21)  25 (40)  4 (19)  4 (44) | 49 (79)  38 (60)  17 (81)  5 (56) | 0.06 |
| CCI  <5  5-7  ≥8 | 54 (22)  63 (18)  38 (16) | 188 (78)  280 (82)  197 (84) | 0.28 | 32 (59)  30 (48)  21 (55) | 22 (41)  33 (52)  17 (45) | 0.44 | 42 (78)  42 (67)  25 (66) | 12 (22)  21 (33)  13 (34) | 0.33 |
| Second Primary Cancer  Yes  No | 2 (3)  153 (20) | 65 (97)  600 (80) | <0.01 | 2 (100)  81 (53) | 0 (0)  72 (47) | 0.18 | 1 (50)  45 (29) | 1 (50)  108 (71) | 0.53 |
| Primary site  Colorectal  Hepatobiliary/Pancreatic  Other | 42 (15)  104 (27)  9 (6) | 247 (85)  285 (73)  134 (94) | <0.01 | 15 (36)  66 (63)  2 (22) | 27 (64)  38 (37)  7 (78) | <0.01 | 5 (12)  40 (38)  1 (11) | 37 (88)  64 (62)  8 (89) | <0.01 |
| AJCC Stage  0/I  II  III  IV | 14 (8)  50 (28)  40 (26)  48 (22) | 161 (92)  130 (72)  111 (74)  173 (78) | <0.01 | 8 (57)  26 (52)  24 (60)  24 (50) | 6 (43)  24 (48)  16 (40)  24 (50) | 0.80 | 8 (57)  38 (76)  26 (65)  34 (71) | 6 (43)  12 (24)  14 (35)  14 (29) | 0.49 |

| Supplemental Table 2: Bivariate Analyses of Trial Eligibility, Documented Offer, and Enrollment, Among Patients with HN Malignancy | | | | | | | | | |
| --- | --- | --- | --- | --- | --- | --- | --- | --- | --- |
|  | Eligibility (N=625) | | | Documented Offer, Among Patients Eligible for Trial (N=150) | | | Enrollment, Among Patients Eligible for Trial (N=150) | | |
| **Characteristic** | **Eligible**  **N(%)** | **Not eligible**  **N(%)** | **P** | **Documented**  **N(%)** | **Not Documented**  **N(%)** | **P** | **Enrolled** | **Not Enrolled** | **P** |
| Gender  Female  Male | 40 (14)  110 (33) | 250 (86)  225 (67) | <0.01 | 7(18)  64(58) | 33(82)  46(42) | <0.01 | 3(8)  41(37) | 37(92)  69(63) | <0.01 |
| Age  <65  65-74  ≥75 | 103(25)  35(24)  12(17) | 317 (75)  98 (76)  60 (83) | <0.01 | 55(53)  14(40)  1(17) | 48(47)  21(60)  10(83) | 0.03 | 35(34)  7(20)  2(17) | 68(66)  28(80)  10(83) | 0.18 |
| Race  White  Black  Asian  Other | 137 (24)  4 (22)  2 (67)  7 (23) | 437 (76)  14 (78)  1 (33)  23 (67) | 0.39 | 64(46)  0(0)  1(50)  6(85) | 73(54)  4(100)  1(50)  1(15) | 0.03 | 40(29)  0(0)  1(50)  3(43) | 97(71)  4(100) 1(50)  4(57) | 0.44 |
| Marital status  Not Married  Married/Partnered | N/A | N/A | - | 21(36)  49(54) | 37(64)  41(46) | 0.03 | 15(26)  29(32) | 43(74)  61(68) | 0.41 |
| Children  0  1 or more | N/A | N/A | - | 7(50)  56(49) | 7(50)  58(51) | 0.95 | 6(43)  35(31) | 8(57)  79(69) | 0.36 |
| Employment status  Full-time  Part-time  Retired  Not working | N/A | N/A | - | 45(64)  0  11(35)  8(47) | 25(36)  0  20(65)  9(53) | <0.01 | 28(40)  0  5(16)  6(34) | 42(60)  0  26(84)  11(66) | 0.03 |
| Primary payer  Private  Medicare  Medicaid  Other | N/A | N/A | - | 33(59)  20(32)  16(57)  0(0) | 23(41)  42(68)  12(43)  2(100) | 0.01 | 21(38)  11(18)  10(36)  0(0) | 35(62)  51(82)  18(64)  2(100) | 0.06 |
| CCI  <5  5-7  ≥8 | 98(25)  45(24)  7(17) | 294(75)  144(76)  35(83) | 0.55 | 50(51)  19(42)  2(29) | 48(49)  26(58)  5(71) | 0.37 | 31(32)  11(24)  2(29) | 67(68)  34(76)  5(71) | 0.68 |
| Second Primary Cancer  Yes  No | 16(12)  134(27) | 119 (88)  356 (73) | <0.01 | 7(44)  64(48) | 9(56)  70(52) | 0.76 | 4(25)  40(30) | 12(75)  94(70) | 0.69 |
| Primary site  Mucosal  Salivary  Thyroid | 143(39)  7(14)  0(0) | 227(61)  42(86)  206(100) | <0.01 | 71(50)  0(0)  0(0) | 72(50)  7(100)  0(0) | 0.01 | 44(31)  0(0)  - | 99(69)  7(100)  - | 0.08 |
| AJCC Stage  0/I  II  III  IV | 2(1)  8(16)  23(28)  117 (47) | 225 (99)  41(84)  59(72)  134(53) | <0.01 | 1(50)  1(13)  11(48)  58(50) | 1(50)  7(87)  12(52)  59(50) | 0.25 | 0(0)  0(0)  4(17)  40(34) | 2(100)  8(100)  19(82)  77(66) | 0.07 |
